# Design and Development of a Rotating Chair to Measure the Cervico-Ocular Reflex

**DOI:** 10.7759/cureus.19099

**Published:** 2021-10-28

**Authors:** Praveen Sanmugananthan, Nam Nguyen, Bernadette Murphy, Ali Hosseini

**Affiliations:** 1 Faculty of Health Sciences, Ontario Tech University, Oshawa, CAN; 2 Faculty of Engineering and Applied Science, Ontario Tech University, Oshawa, CAN

**Keywords:** cor, subclinical neck pain, gaze stabilization, sensorimotor integration, cervico-ocular reflex

## Abstract

Eye reflexes that stabilize gaze are essential in navigating daily life. One such reflex is the cervico-ocular reflex (COR). An important neural structure involved in the COR is the cerebellum, which facilitates proper gaze stability through sensorimotor integration to adjust eye movements accordingly. This reflex is tested by fixating the head in space and rotating the body around the neck. Thus, a rotating chair is needed to elicit proper cervical rotation while keeping the head fixed. The chair that was developed for this project was able to rotate to the specified amplitude (±0.5º of accuracy) and frequency. The parameters of the rotation amount, frequency, and amplitude can be adjusted as desired by the project guidelines. Our project aimed to improve upon existing chair models and develop a chair that can be used to assess the COR in neck pain populations.

## Introduction

The cervico-ocular reflex (COR) is a reflexive eye movement that stabilizes gaze during trunk rotation [1]. Sensory information from cervical nerves relay proprioceptive information to the cerebellum via vestibular nuclei in the brainstem, resulting in eye movements that oppose the head movements [2,3]. These eye movements are governed by cranial nerves (CN) III, IV, and VI [4]. The vast majority of ocular movements are innervated by the oculomotor nerve (CN III); however, lateral eye movements controlled by the lateral rectus muscle which is innervated by the abducens nerve (CN VI) is important for COR rotation since the rotation of the chair is in the transverse plane [4]. However, their central regulation, as mentioned, is via vestibular nuclei and the cerebellum [2,3]. To elicit COR, the head is fixated in space and the trunk is rotated about the neck. In past studies, rotation was performed in absolute darkness to eliminate input from other sensory modalities, such as vision and the vestibular-ocular reflex (VOR) [2]. The data collected from the current studies is a series of eye movements in response to the rotation of the chair. This can give insight into how the CNS incorporates sensory information in order to stabilize gaze and whether this ability is impacted by experimental perturbations or in specific groups such as those with neck pain. COR gain is measured by dividing the eye velocity by the velocity of the chair [1]. Blinks, saccades, and fast phases are removed, and a sine fit of the eye data is created [1]. This provides a metric that illustrates how much the eye has to move to compensate for trunk rotation. The COR is most responsive during low-frequency rotations [2], and it is optimally induced at a 5º rotation amplitude at a frequency of 0.04 Hz [1].

Research investigating the COR is growing, thus it is imperative to develop a chair that is appropriate for the research population being studied. Prior studies have utilized various methods to stabilize the head during trunk rotation. For example, one study had the researcher hold the participant’s head still during trunk rotation [5]. Furthermore, a laser apparatus was attached to the head of the participant to illustrate the visual target. This setup was not optimal for our research as we needed to monitor eye movement using high-speed eye-tracking software EyeLink II (SR Research, Ottawa, ON) integrated with a 3D Investigator motion capture system to track trunk movement, where an experimenter’s hands could get in the way of the motion capture cameras. Therefore, it was important to fix the head to the chair during the rotations. Prior studies have also utilized a bite board, where participants bit down to fixate the head during rotation [1,2,6]. This method is not ideal for neck pain participants as biting down activates the supra- and infra-hyoid muscles located bilaterally on the side of the neck [7]. Constant activation of these muscles during the protocol may result in fatigue, which may worsen the neck pain condition. Therefore, a rotation chair is needed which can fix the head position without the need for assistance, while avoiding the use of a bite board. The purpose of this technical report is to outline the processes involved in developing and operating the rotation chair developed to induce the COR.

## Technical report

Conditions, inputs, process, and outcome

The chair was designed to measure the COR in those with subclinical neck pain (SCNP) in order to determine how cerebellar deficits previously observed in this population impact ocular movements such as the COR [8]. Experimenters will be operating the COR chair that requires a user interface (UI) along with 3D motion capture cameras and an eye-tracking apparatus that uses Experiment Builder software (SR Research, Ottawa, ON) to run the program. The COR is measured by collecting eye data while the head is fixated during trunk rotation. Each participant will perform 25 rotations in total, including five rotations per trial for five trials. Eye data is measured using the Eyelink II eye-tracking device (SR Research, Ottawa, ON) while COR rotation amplitude and velocity are measured using 3D motion capture system.

Participants

Operation of the chair requires trained experimenters that have ethical approval to conduct research involving humans. Participants can be anyone that are appropriate for the parameters of the study (i.e., investigating special populations and/or healthy controls). Participants are informed about the nature of the research and sign a written consent prior to the study.

Setting and equipment 

The COR chair is a custom-designed chair made by the research team at Ontario Tech University, Oshawa, Canada. The materials used to build the chair were available off-the-shelf components, which also allowed us to easily make any modifications or adjustments.

Specifications

**Figure 1:**
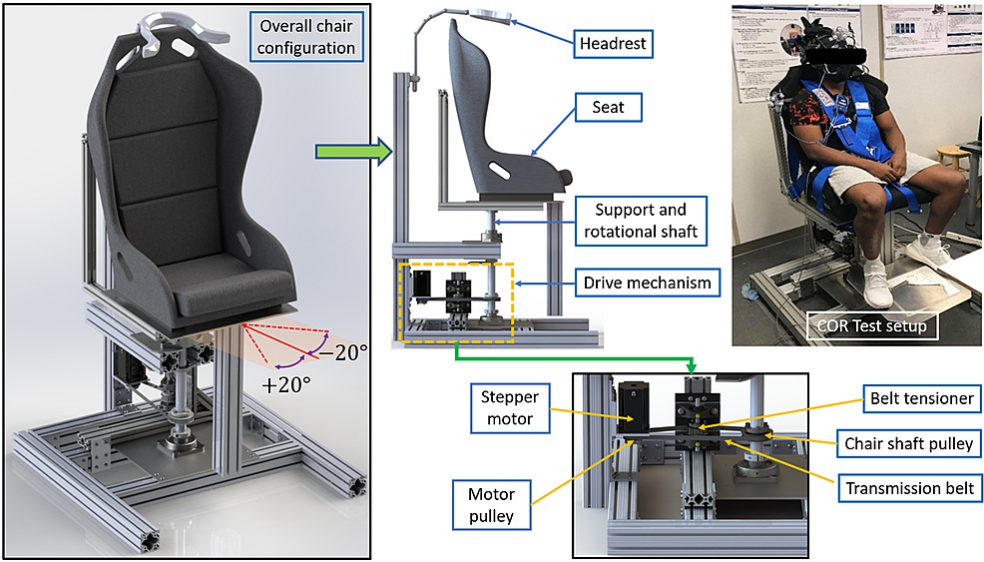
COR chair configuration and components COR: cervico-ocular reflex

The chair structure was built using T-slotted framing aluminum extrusion (Figure 1). The overall dimensions of the chair are 0.91 m (36″) in length, 0.91 m (36″) in width, and 1.8 m (70″) in height. The chair worked as expected with test subjects of varying weights. The chair has a rotational limit of -20º (left) to +20º (right) in the horizontal plane. Rotational accuracy is within ±0.5º tolerance. Based on the stepper motor driver setting of 7.2 A peak current and output voltage of 48 VDC, the peak power consumption is approximately 350 W.

Components and Functions

The chair mechanical system consists of aluminum structural frame, pulley system, belt and tensioner, chair, safety harness, and headrest (Figure 1). The majority of the structural frame was built using quad 3″ by 3″ T-slot framing extrusion rails. T-slot rails allow other components to be fastened at any location along rails’ length. The entire weight of the seat is supported by a 1.5″ diameter solid steel shaft, with bearing and bearing sleeve bolted to the base at the bottom of the shaft. This shaft also transfers rotation from motor to the seat. Additional quad rails are used to hold the shaft vertically. A timing belt is used to transfer rotation from motor to shaft. Some sags are required to install the belt so that it dramatically reduces rotational accuracy. A belt tensioning system was later installed to eliminate this issue. The seat used is an NRG bucket seat. A five-point harness is used to fasten the subject’s body to the seat. The headrest is located above the seat, used to keep the head stationary while the body rotates with the seat. The pulley system results in a gearing ratio of 1.56:1, meaning that the seat will turn 1º for every 1.56º turn of the motor shaft.

**Figure 2:**
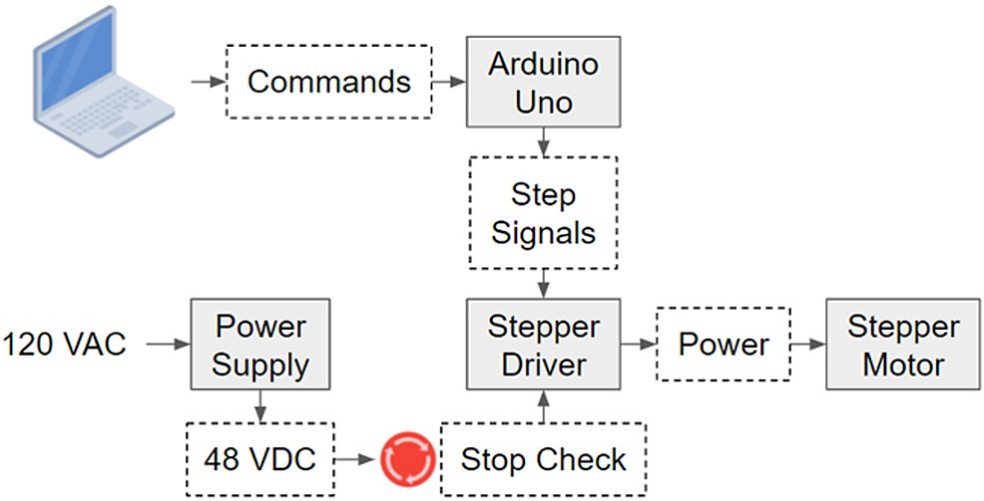
COR chair control diagram COR: cervico-ocular reflex

The electrical system consists of stepper motor, motor driver, power supply, and Arduino Uno (Figure 2). The default step resolution of the stepper motor is 1.8º/step. The stepper driver is set to 1/32 microstepping, and in addition with the gearing ratio of 1.56:1, the seat rotational resolution is improved to 0.036º. However, rotational accuracy is ±0.5º due to the elasticity of the timing belt. The stepper motor is a SureStep NEMA 34, which is capable of outputting a maximum of 9 Nm of torque. With the pulley ratio of 1.56:1, the maximum torque output to the seat is 14 Nm. The motor driver used is a MA860H stepper driver, with peak current output is 7.2 A (5.1 A RMS). The power supply converts 120 VAC to 48 VDC for the stepper driver. The Arduino Uno receives instructions from the connected computer, converts the instructions into pulse-width modulation (PWM) signals, and sends the signals to the stepper driver, which drives the motor in steps. Two emergency stop switches are hard-wired between the power supply and the stepper driver. These stop buttons cut off power to the driver and hence de-energize motors in case of problems.

**Figure 3:**
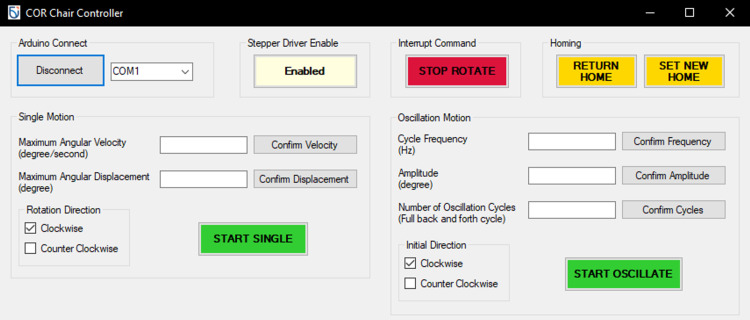
COR chair control user interface COR: cervico-ocular reflex

The software required are Arduino IDE version 1.8.16 (Arduino, Somerville, MA) and COR Chair Controller UI. Arduino IDE is used to program the Arduino Uno to receive commands from COR chair controller UI through USB serial port, and then translate the commands into signals to the stepper driver. The UI application allows the operator to quickly define a rotational parameter and initiate it (Figure 3). The UI application supports single rotation, or oscillation rotation, created using Visual Studio Form.

**Figure 4:**
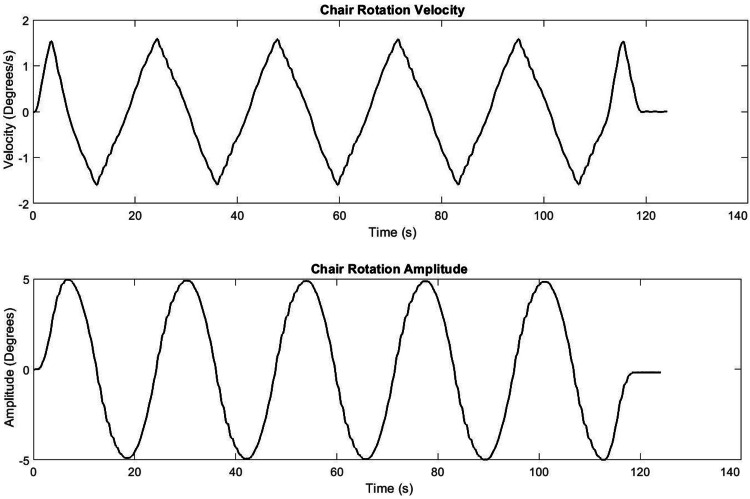
COR chair angular velocity and position profile COR: cervico-ocular reflex

Oscillation rotation is defined using cycle frequency, amplitude, and number of cycles. Oscillation starts in the center, rotates to negative amplitude (left), then to positive amplitude (right). Each cycle is defined as completing one negative amplitude, one positive amplitude, and then returning to the center at 0º. Figure 4 below shows the resulting angular position and angular velocity profile of an oscillation test at 0.05 Hz and rotational amplitude of 5º.

Scenario template

A rigid body containing three infrared light-emitting diodes (IRED) markers is attached to the right shoulder area of the chair such that the participant’s shoulder will not impede the visibility of the rigid body. Motion capture camera setup is completed prior to the arrival of the participant. The cameras collect at a frequency of 100 Hz for 120 seconds (the duration of each trial).

The participant is secured with safety belts around each shoulder and the waist. The eye tracker is placed on the participant’s head to measure eye movements and head is fixed to the headrest using a headband to avoid rotation. The target apparatus appears on a screen roughly 160 cm from the chair using Experiment Builder software. Once the eye cameras are adjusted, the researcher sets the parameters of chair rotation using the UI.

The protocol involves 25 rotations split evenly between five trials (five rotations per trial) at an amplitude of 5º with a frequency of 0.04 Hz [1]. Prior to the start of each trial, a screen appears depicting a small target intended to correct for eye drift. Initiation of the trial begins once the researcher hits enter on the keyboard. A yellow target appears briefly for 3 seconds before disappearing for the remainder of the trial. Participants are instructed to stare at the target and where they last saw the target for the remainder of the trial. The first trial begins rotation in the clockwise direction, and alternates between clockwise and counter clockwise for the remainder of the trials.

Supplemental materials

The Optotrak 3D Investigator™ motion capture system (Northern Digital Inc., Waterloo, ON) was used to validate the accuracy of the seat rotation. A rigid body consisting of three IRED markers was placed on the seat and tracked using the Optotrak 3D cameras. After positional data has been collected, MATLAB software (MathWorks, Natick, MA) was used to calculate the rotation and velocity of the chair. The Eyelink II eye-tracking device is used to track the movement of the eyes during rotation. Eye data is analyzed using custom MATLAB code.

## Discussion

The purpose of this technical report was to provide a comprehensive outline for the creation, implementation, and validation of the rotating chair necessary to test the COR. Preliminary test data show that the chair is able to rotate to the specified amplitude within ±0.5º accuracy. Accurate rotation of the chair is necessary as prior studies have illustrated that the optimal parameters to induce COR require low rotation amplitude and frequency [1]. In addition, this technical report sought to develop a chair that would reduce the discomfort to the patients with neck pain. Prior studies have utilized bite boards which may exacerbate neck pain [1,2,6]. These studies found that the bite board apparatus provided negligible head rotation [1,9]. The current chair utilizes a headrest in which the head can be strapped to limit neck pain and the degree of head movement. Head rotation was tested using 3D motion capture system and was also found to be negligibly small. Furthermore, the efficacy of this chair was tested on lab members with neck pain. Subjective verbal ratings of these lab members suggested that the chair rotation did not make their neck pain worse. Prior studies did not mention the rotational accuracy of chairs. The current chair was designed with low rotational amplitude in mind, thus requiring stricter accuracy settings which were achieved within 0.5º. The benefit of such a tight window of rotational accuracy is that it allows for precise measurement of CNS impairment.

Furthermore, the addition of the UI allows for the parameters of the rotation amplitude and frequency to be changed to match the parameters set at the beginning of the research study. This provides additional benefit to seamlessly test the impact of varying frequencies on the COR on a trial-to-trial basis. For example, this can be incorporated into future studies testing a participant’s ability for COR adaption to changing stimuli (i.e., via rotation velocity) and how this is impacted in populations with altered sensorimotor integration. In addition, this can be incorporated into future studies that can test passive VOR or trunk proprioception. For example, prior studies have tested VOR using both passive and active head rotations [10,11]. In these studies, active rotation is defined by rotations performed by the subject while passive is conducted by the experimenter. VOR is elicited by rotating the head while fixating on a target, thus resulting in an eye movement in the opposite direction of head movement [10]. Therefore, instead of fixing the head in space, the participant’s head can be in line with their body axis to allow for passive manipulation of VOR by using the chair. Prior studies have also investigated trunk proprioception using a rotating chair [12]. Proprioception involves the relay of afferent sensory information responsible for kinesthetic awareness [13]. To investigate trunk proprioception, the participant is rotated in the absence of vision and must recreate the angle in which they rotated. Therefore, this rotating chair can not only be utilized to test COR function but also to test passive versus active VOR and trunk proprioception in special populations.

Some limitations include a slight jerk in the movement during chair deceleration. As mentioned previously, some unanticipated outcomes could be a large shift in amplitude due to the movement of the participant during collection. These shifts in amplitude present in a noticeable atypical shift in the amplitude graph. To avoid this, emphasize to the participant that they must remain still throughout the rotation of the chair to avoid large shifts in rotational amplitude.

## Conclusions

The designed chair was able to function adequately to allow collection of COR data which is important for investigating cerebellar deficits in special populations and in response to experimental perturbations. This design represented a low-cost and open-sourced solution for COR data collection and research, an alternative to the limited and costly options currently available in the medical equipment market. Furthermore, this design accommodates for those with neck pain and mitigates the risk for neck fatigue while performing the study. Future redesign and development can be based on this first COR chair iteration, where rotational accuracy and stability can be improved further.
